# Identification and comparison of m6A modifications in glioblastoma non-coding RNAs with MeRIP-seq and Nanopore dRNA-seq

**DOI:** 10.1080/15592294.2022.2163365

**Published:** 2023-01-03

**Authors:** Raulas Krusnauskas, Rytis Stakaitis, Giedrius Steponaitis, Kristian Almstrup, Paulina Vaitkiene

**Affiliations:** a Laboratory of Molecular Neurobiology, Neuroscience Institute, Lithuanian University of Health Sciences, Eiveniu str. 4, LT50161, Kaunas, Lithuania; b Laboratory of Molecular Neurooncology, Neuroscience Institute, Lithuanian University of Health Sciences, Eiveniu str. 4, LT50161, Kaunas, Lithuania; c Department of Growth and Reproduction, Rigshospitalet, University of Copenhagen, GR-5064, Rigshospitalet, Blegdamsvej 9, DK-2100 Copenhagen, Denmark; d International Center for Research and Research Training in Endocrine Disruption of Male Reproduction and Child Health (Edmarc), Rigshospitalet, University of Copenhagen, GR-5064, Rigshospitalet, Blegdamsvej 9, DK-2100 Copenhagen, Denmark

**Keywords:** lncRNA, m6A, epi-transcriptome, glioblastoma, MeRIP-seq, nanopore dRNA-seq

## Abstract

The most prominent RNA modification – N6-methyladenosine (m6A) – affects gene regulation and cancer progression. The extent and effect of m6A on long non-coding RNAs (lncRNAs) is, however, still not clear. The most established method for m6A detection is methylated RNA immunoprecipitation and sequencing (MeRIP-seq). However, Oxford Nanopore Technologies recently developed direct RNA-seq (dRNA-seq) method, allowing m6A identification at higher resolution and in its native form. We performed whole transcriptome sequencing of the glioblastoma cell line U87-MG with both MeRIP-seq and dRNA-seq. For MeRIP-seq, m6A peaks were identified using nf-core/chipseq, and for dRNA-seq – EpiNano pipeline. MeRIP-seq analysis revealed *5086* lncRNAs transcripts, while dRNA-seq identified 336 lncRNAs transcripts from which 556 and 198 were found to be m6A modified, respectively. While 24 lncRNAs with m6A overlapped between two methods. Gliovis database analysis revealed that the expression of the major part of identified overlapping lncRNAs was associated with glioma grade or patient survival prognosis. We found that the frequency of m6A occurrence in lncRNAs varied more than 9-fold throughout the provided list of 24 modified lncRNAs. The highest m6A frequency was detected in *MIR1915HG, THAP9-AS1, MALAT1, NORAD1*, and *NEAT1 (*49–88nt), while *MIR99AHG, SNHG3, LOXL1-AS1, ILF3-DT* showed the lowest m6A frequency (445–261nt). Taken together, (1) we provide a high accuracy list of 24 m6A modified lncRNAs of U87-MG cells; (2) we conclude that MeRIP-seq is more suitable for an initial m6A screening study, due to its higher lncRNA coverage, whereas dRNA-seq is most useful when more in-depth analysis of m6A quantity and precise location is of interest.

**Abbreviations:** (dRNA-seq) direct RNA-seq, (GBM) glioblastoma, (LGG) low-grade glioma, (lncRNAs) long non-coding RNAs, (m6A) N6-methyladenosine, (MeRIP-seq) methylated RNA immunoprecipitation and sequencing, (ncRNA) non-coding RNA, (ONT) Oxford Nanopore Technologi; Lietuvos Mokslo Taryba

## Background

First chemical RNA modification was discovered by Waldo E. Cohn and Elliot Volkin in 1951 [1]. Almost 70 years later, this knowledge expanded to more than 150 identified RNA modifications [2]. Interestingly, the field of epi-transcriptomics recently regained its interest due to characterization of the regulatory machinery of *N*6-methyladenosine (m6A) modification [3,4] and a breakthrough of its efficient recognition methods [5,6]. *N*6-methyladenosine is a reversibly methylated adenosine, which is co-/post-transcriptionally installed by ‘writers’ [7], interpreted by ‘readers’ [8], and removed by ‘erasers’ [4,9]. Thorough mapping and identification of specific RNA molecules containing m6A revealed modification importance and role in gene expression [10], development [11], and disease progression [12]. Recent development of novel approaches for m6A identification revealed limitations of the first-generation methods and highlighted directions for further improvements, primarily related to increased registration sensitivity and bioinformatics [13]. Furthermore, majority of the studies solely focuses on the methylation of coding RNAs, while ignoring hugely important and m6A modified non-coding RNA (ncRNA) molecules [14].

Human genome contains over 16,000 genes encoding long non-coding RNAs (lncRNAs) (Gencode v30) which leads to production of more than 30,000 transcripts [15]. Long non-coding RNAs (lncRNAs) interact with DNA, RNA, and proteins [16–19], and by doing so affect gene regulation, proliferation, infiltration, and metastasis of tumour cells. Methylated RNA immunoprecipitation and sequencing (MeRIP-seq) and m6A iCLIP (miCLIP) experiments revealed that *XIST* lncRNAs contain 78 m6A marks [20]. Later, Patil et al. showed that m6A is crucial for *XIST* functionality which was supported by interactions with key components of m6A machinery [21]. Another lncRNA, metastasis-associated lung adenocarcinoma transcript 1 (*MALAT1*) also contains high level of m6A modifications [22]. Furthermore, *MALAT1* m6As not only affect its cellular location and binding properties but also change its structure [22].

Currently, m6A is primarily recognized by MeRIP-seq method based on anti-m6A antibodies [5,6]. These antibodies specifically bind to methylated adenosines and enable identification of the approximate location of modifications. More recently, identification of RNA modifications was introduced by Oxford Nanopore Technologies (ONT) direct RNA-seq (dRNA-seq) method [13]. dRNA-seq enables precise detection of RNA modifications including m6A in its native form, by pulling molecules through the membrane containing embedded nanopore particles. ONT sequencing device registers nucleotides by measuring disruptions in the electric current intensity as an RNA molecule passes through the pore. Even though dRNA-seq is an established method which can register RNA modifications at a single nucleotide resolution, its efficiency and accuracy require further improvements [13].

The aim of the study was (1) to define a list of m6A locations in lncRNAs of U87-MG cell line; and (2) to compare MeRIP-seq and dRNA-seq methods for m6A detection in lncRNAs. We hypothesized that dRNA-seq would result in a more precise profile of lncRNA epi-transcriptome in U87-MG cells, compared to MeRIP-seq. And that molecules identified by both methods could be used in future glioma treatment or screening studies.

## Materials and methods

### Experimental design of sequencing experiments

**Figure 1.**
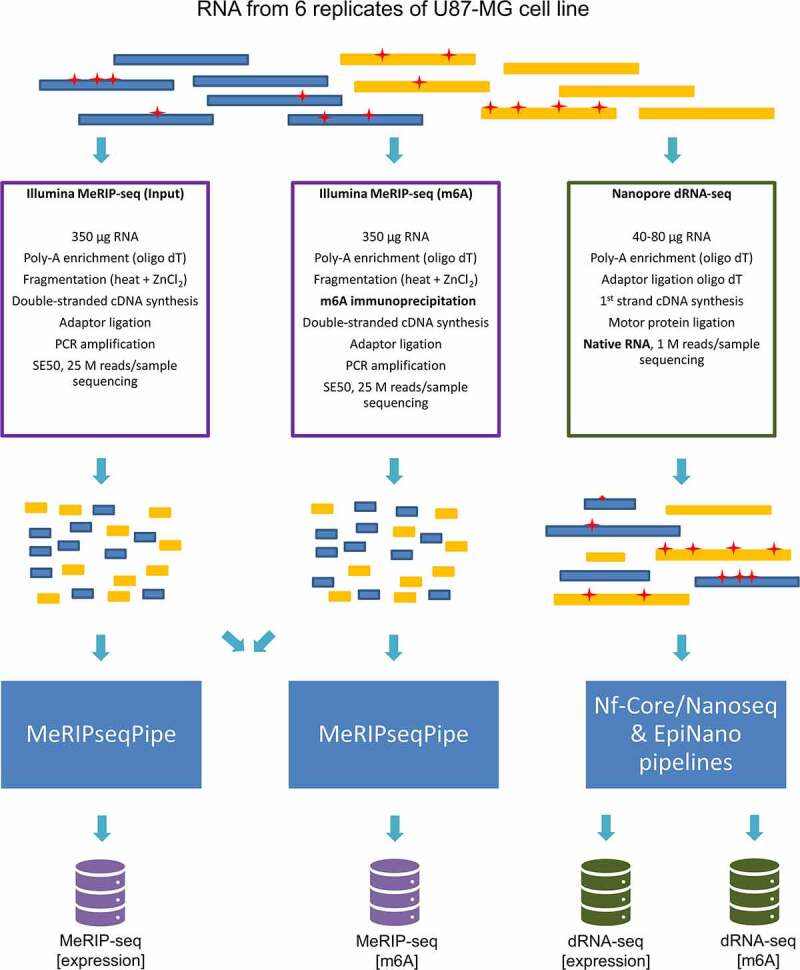
Experimental design of MeRIP-seq and dRNA-seq of U87-MG cells. The coloured blocks indicate poly-adenylated RNA molecules that are being analysed. Red asterisks represent m6A modifications in RNA molecules. A native RNA molecule that has retained RNA modifications is analysed by dRNA-seq, while MeRIP-seq is used to analyse short cDNA products and data from two libraries (before and after immunoprecipitation) are compared.

For both platforms, two replicates (R1 and R2) of U87-MG cell lines were used. In total 6 replicates were obtained: two for MeRIP-seq [expression] (input), two for MeRIP-seq [m6A] (m6A) and two for dRNA-seq (as it does not require input dataset, expression and m6A information is obtained from the same sample. However separate pipelines were applied for the evaluation of lncRNA expression and m6A modification (following indicated as [expression] and [m6A], respectively)). Experimental design and key differences in library preparation are depicted in (Figure 1). MeRIP-seq requires cDNA synthesis and PCR amplification, which results in sequencing of PCR amplicon. On the other hand, dRNA-seq employs 1st strand cDNA synthesis to ensure molecule stability, resulting in sequencing of native RNA. More detailed comparison of the two methods is presented in **Table S1**.

### Cell culture

Human glioblastoma U87-MG cells were obtained from the European Collection of Cell Cultures (ECACC, cat. No. 89,081,402). Humidified incubator with 5% CO_2_ at 37°C was used for cell culture. Mycoplasma contamination was routinely assessed [23]. Cells were cultured in a high glucose DMEM media (Gibco, cat. No. 10,566,016) supplemented with 10% foetal bovine serum (Gibco, cat. No. 10,500,064), 100 IU/mL penicillin, and 100 µg/mL streptomycin (Gibco, cat. No. 15,140,122). Cells were passaged with trypsin (Gibco, Cat. No. 25,300,062) upon reaching 80–90% confluency. A total of 15–20 plates, each containing 3 million cells (13th passage), were used for RNA extraction. Replicate samples (R1 and R2) contain RNA pooled from at least three different plates.

### RNA extraction and polyA RNA enrichment

Culturing media was replaced with ice-cold PBS (Phosphate Buffered Saline). Cells were scrapped, collected, centrifuged at 180 x g for 5 min at 4°C, and stored at −80°C. Lysis was performed with Trizol (Invitrogen, cat. No. 15,596,026) following manufacturer’s protocol. Extracted RNA was quantified by nanodrop and stored at −80°C. Extracted RNA (± 700 mg) was subjected to polyA RNA enrichment with DynaBeads (Invitrogen, cat. No. 61,012) following the manufacturer’s protocol. PolyA enriched RNA was precipitated overnight, resuspended in nuclease free water, and stored at −80°C. RNA quality and quantity were assessed by Agilent’s Bioanalyzer (Agilent Technologies, cat. No. 5067–1513) and Nanodrop spectrophotometer (Thermo Fisher Scientific, cat. No. ND-2000).

### Methylated RNA immunoprecipitation (MeRIP)

Dominissini and Meyer (2012) protocols were followed [5,6]. 18 mg of polyA enriched RNA was mixed with fragmentation buffer (Sigma Aldrich, cat. No. 17–10,499), heated for 3 min at 94°C. RNA was placed on ice, mixed with 2 mL 0.5 M EDTA, and precipitated in ethanol overnight. 10 μg of m6A antibodies (SySy, cat. No. 202 111) were mixed with 250 ng of magnetic beads (ThermoFisher Scientific, cat. No. 88,803) in freshly made IP buffer (50 mM Tris-HCl 7.5 pH, 150 mM NaCl, 0.1% (vol/vol)) for 6 h at 4°C. Washed complexes were mixed with 5 mg of prepared RNA in 500 mL IP buffer supplemented with RNase inhibitors (final conc. 0.3 U/μL) (Invitrogen, cat. No. AM2694) and RVC at final concentration of 2 mM (Sigma Aldrich, cat. No. R3380). RNA with beads were incubated overnight at 4°C. Complex was washed twice with IP buffer supplemented with RNase inhibitors (final conc. 0.1 U/μL) (Invitrogen, cat. No. AM2694) and once with high salt wash buffer (50 mM Tris-HCl 7.5 pH, 300 mM NaCl, 0.1% (vol/vol) with RNase inhibitors (final conc. 0.1 U/μL) (Invitrogen, cat. No. AM2694)). RNA was eluted from beads with 100 μL elution buffer (IP buffer supplemented with a N6-Methyladenosine, 5'-monophosphate sodium salt (Merk, cat. No. CS220007) (final concentration of 6.7 mM), and RNase inhibitors (final conc. 0.1 U/μL) (Invitrogen, cat. No. AM2694) incubating samples on a rotor for 1 h at 4°C. The procedure was repeated twice. Eluted RNA was precipitated overnight and stored at −80°C.

### MeRIP-seq and data analysis

MeRIP-seq RNA quality and quantity were assessed by Agilent’s Bioanalyzer (Agilent Technologies, cat. No. 5067–1513) and Qubit RNA HS kit (Invitrogen, cat. No. Q32852). MeRIP-seq libraries were prepared with Novogene’s RIP-seq protocol, which (i) synthesized double-stranded cDNA, (ii) ligated sequencing adapters and polyA to 5’ and 3’ respectively, and (iii) amplified the libraries with PCR. After library purification and quantification, a single-end 50 bp sequencing was performed on Illumina platform (minimum of 25 M reads/sample). Gene/transcript quantification and m6A calling were computed running nf-core/rnaseq and nf-core/chipseq pipelines, respectively [24]. Non-stranded reads were aligned to Ensembl human reference genome (GRCh38.p13.105) using hisat2 [25]. Further analysis was carried out with default parameters.

### Nanopore direct RNA sequencing

PolyA enriched RNA of U87-MG was used for dRNA-seq. Three extracts of U87-MG polyA RNA were mixed to generate homogeneous sample for sequencing. 800–1000 ng of RNA was used to generate nanopore libraries following the manufacturers protocol (Oxford Nanopore Technologies, cat. No. SQK-RNA002, ver.: 6.0.7). Purified libraries were quantified with Qubit dsDNA HS kit (Invitrogen, cat. No. Q32851); At least 80–100 ng of prepared libraries were used for sequencing. A MinION device (Oxford Nanopore Technologies, cat. No. Mk1B) and flow cells (Oxford Nanopore Technologies, cat. No. FLO-MIN106D) were used for sequencing. Manufacturer’s instructions were followed for all procedures. All experiments were carried out with a flow cells containing 1200–1500 live pores. Sequencing ran for 48–54 h.

### Nanopore dRNA-seq data analysis

Signal detection was done by MinKNOW software (ver.: 4.0.4). Guppy (ver.: 4.0.9 + 92ae093) was used for base-calling with high accuracy option on ubuntu 20.04 computer system. Gene/transcript quantification performed with nf-core/nanoseq pipeline [26]. Alignments were made against Ensembl’s GRCh38 reference genome. For detection of m6A modification, the following steps were made: (i) sequencing reads were base called with Guppy, (ii) alignment were made with Minimap2 [27] (ver.: 2.20-r1061) (*minimap2 – axe splice – uf -k14*) to Ensembl’s GRCh38 reference transcriptome (cDNA + ncRNA), (iii) filtering, sorting, and indexing mapped reads with SAMtools [28] (ver.: 1.12) (*samtools view -bS -F 4 – u -q 2*), (iv) m6A modification prediction with EpiNano [13] (ver.: 1.2) within 5-mer RRACH motifs.

### QC analysis

Post-alignment quality was evaluated applying SAMtools (*samtools flagstat*) [24]. Picard (*picard CollectRnaSeqMetrics*), RSeQC [29,30]] (*read_distribution.py; bam_stat.py; read_GC.py*), and NanoStat [31] softwares.

### Bioinformatic analysis in glioma samples

Transcript levels of lncRNA genes in patients’ samples of Glioblastoma (GBM), Low-grade glioma (LGG) and normal brain were obtained from GlioVis data portal [32]. LncRNA expression association with GBM grades and overall survival were analysed. lncRNA expression differences between pairs p‑values were determined using Tukey’s Honest Significant Difference. Brain tumor datasets visualisation tool *GlioVis* was used for Kaplan–Meier analysis of overall survival data of Glioma patients based on the lncRNA expression levels. The log-rank test was used to calculate the p-values for the significance of the difference between the high and low expression of lncRNA.

## Results

### Quality control information of overall RNAs and only lncRNAs

**Figure 2.**
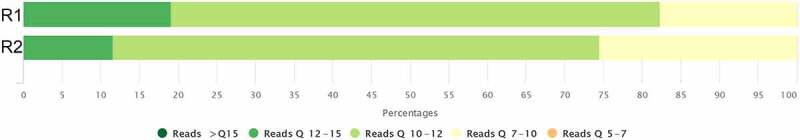
dRNA-seq read quality distribution of all reads. R1 and R2 represent two replicates of dRNA-seq. Q – reads quality.

MeRIP-seq and dRNA-seq are powerful methods that provide a quantitative and informative view of the transcriptome with the number of sequenced molecules being a key element in transcriptome analysis. dRNA-seq resulted in 0.7 and 0.9 M reads for replicate 1 and 2. Median quality of dRNA-seq was ~11Q, meaning that the base call accuracy was ~90%. dRNA-seq replicates resulted in similar quality of read distribution with slightly higher quality reads obtained in the first replicate (Figure 2). Interestingly the first replicate resulted in longer N50 read when compared to the second replicate (Table 1). MeRIP-seq produced 50 bp reads, which did not differ between replicates due to sequencing design. First and second m6A MeRIP-seq replicates had 39.8 and 38 M reads of high quality >35.9Q, indicating that the probability of an incorrect base call was less than 1 out of 1000. The read quality measurements of both approaches are usual and expected for both platforms.

**Table 1.** MeRIP-seq and dRNA-seq quality control results of all reads.

Sample name	Method	M reads mapped	Median Quality	Median length, bp	N50
MeRIP-seq [m6A]_R1	MeRIP-seq	39.8	37	50	-
MeRIP-seq [m6A] _R2	MeRIP-seq	38	35.9	50	-
MeRIP-seq [expression]_R1	MeRIP-seq	70.9	35.9	50	-
MeRIP-seq [expression]_R2	MeRIP-seq	101.3	37	50	-
dRNA-seq_R1	dRNA-seq	0.7	11.1	973	1605
dRNA-seq_R2	dRNA-seq	0.9	10.7	772	1185

**Figure 3.**
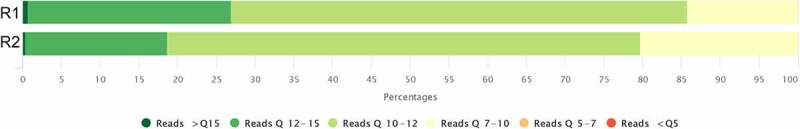
lncRNA read quality distribution in dRNA-seq. R1 and R2 represent two replicates of dRNA-seq. Q – reads quality.

To investigate potential MeRIP-seq or dRNA-seq suitability for m6A identification in lncRNAs, we decided to evaluate quality control (QC) parameters specifically to lncRNAs (Table 2 **and** Figure 3). To this end we used custom pipeline for lncRNAs alignments and QC. Median quality of lncRNA reads obtained by dRNA-seq was ~11Q, and MeRIP-seq lncRNA is ~36Q (Table 2). This indicates that read quality is not affected by molecules biotype in any of the sequencing methods. The N50 read and read length distribution was similar in both lncRNA-alone and overall RNA analyses.

**Table 2.** MeRIP-seq and dRNA-seq quality control results of lncRNA reads.

Sample name	Method	M reads mapped	Median Qual	Median length, bp	N50
MeRIP-seq [m6A]_R1	MeRIP-seq	4.7	35.9	50	-
MeRIP-seq [m6A] _R2	MeRIP-seq	9.1	35.9	50	-
MeRIP-seq [expression]_R1	MeRIP-seq	12.4	35.9	50	-
MeRIP-seq [expression]_R2	MeRIP-seq	17.6	35.9	50	-
dRNA-seq_R1	dRNA-seq	0.8	11.3	741	1448
dRNA-seq_R2	dRNA-seq	0.9	11	661	1095

### Detection of lncRNA genes in U87-MG cell line using MeRIP-seq and Nanopore dRNA-seq platforms

**Figure 4.**
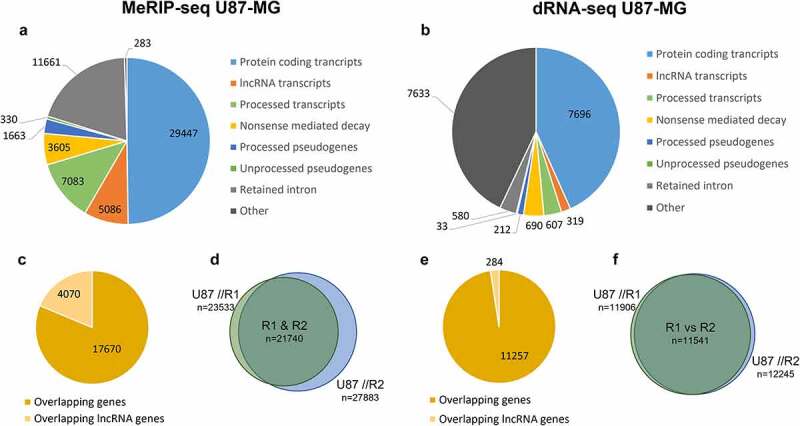
Summary of MeRIP-seq [expression] and dRNA-seq reads. The composition of the sequenced transcripts from MeRIP-seq [expression] (a) and dRNA-seq (b). The composition of sequenced overlapping non-lncRNA genes and lncRNA genes is illustrated in panel (c) and (e) from MeRIP-seq [expression] and dRNA-seq, respectively. Venn diagrams in panels (d) and (f) illustrate the overlap between sequenced genes of R1 and R2 replicates from MeRIP-seq [expression] and dRNA-seq respectively. The figure is constructed based on Input samples from MeRIP-seq and.

Both biological replicates (R1 and R2) were used for MeRIP-seq and dRNA-seq analysis; raw sequencing data was processed through the same pipelines, as described in the methods section (for MeRIP-seq and dRNA-seq accordingly). The final datasets, representing MeRIP-seq and dRNA-seq, were obtained by combining the processed sequencing data of both replicates and filtering out non-overlapping genes/transcripts (Figure 4). In total MeRIP-seq [expression] identified 23,533 and 27,883 genes from each replicate, of which 4617 and 6262 were assigned to lncRNAs. Between replicates 4070 lncRNAs were conserved (Figure 4c). Meanwhile, the dRNA-seq [expression] analysis resulted in 11,906 and 12,245 genes from each replicate, of which 312 and 334 were assigned to lncRNAs; 284 genes overlapped between replicates (Figure 4e).

A summary of MeRIP-seq and dRNA-seq data of the two U87-MG replicates (at gene level) is provided in (Figure 4d, f). MeRIP-seq [expression] identified ~14 times more lncRNA genes than dRNA-seq, which might be associated with higher reads obtained from MeRIP-seq, and PCR amplification step involved in its library preparation, which enables capture of lowly expressed lncRNA molecules.

### Detection of m6A modifications on lncRNAs in the U87-MG cell line using MeRIP-seq and nanopore dRNA-seq technologies

The overall experimental design resulted in 4 different datasets:

1. Gene expression from MeRIP-seq (MeRIP-seq [expression]),

2. m6A peaks from MeRIP-seq (MeRIP-seq [m6A]),

3. Gene expression from Nanopore dRNA-seq (dRNA-seq [expression]),

4. m6A modifications from Nanopore dRNA-seq (dRNA-seq [m6A]).

In the MeRIP-seq [expression] dataset 5086 lncRNAs transcripts were identified. m6A analysis revealed that ~10.9% of detected lncRNAs had modification. While dRNA-seq [expression] database contained 336 lncRNA transcripts, from which ~58.9% were m6A modified. MeRIP-seq and dRNA-seq analysis respectively revealed 556 and 198 m6A modified lncRNA transcripts (Table 3). In conclusion, MeRIP-seq identified ~2.8 times more m6A modified lncRNAs. We decided to combine all 4 datasets to obtain a list of m6A modified lncRNA transcripts which expression and m6A modification status have been confirmed by both sequencing platforms (Table 3 **and** 4). dRNA-seq and MeRIP-seq methods identified 24 overlapping lncRNA molecules with m6A modifications in the U87-MG cell line (Table 4).

**Table 3.** Quantities of overlapping lncRNA molecules (transcripts), identified by MeRIP-seq and dRNA-seq.

	MeRIP-seq [expression]	MeRIP-seq [m6A]	dRNA-seq [expression]	dRNA-seq [m6A]
MeRIP-seq [expression]	5086	556*	127	105* /170#
MeRIP-seq [m6A]		999*	41	24* /33#
dRNA-seq [expression]			319	49* /63#
dRNA-seq [m6A]				198* /336#

The asterix ‘*’ indicates number of lncRNAs having m6A methylated peaks from MeRIP-seq or methylated RRACH motifs from dRNA-seq. The hashtag ‘#’ indicates overall number of lncRNAs for which RRACH motifs were detected and analysed applying EpiNano pipeline.

**Table 4.** List of lncRNA which m6A modifications that were identified both by MeRIP-seq and dRNA-seq methods.

Ensembl Gene ID	Gene name	MeRIP-seq		dRNA-seq (Nanopore)				Association with glioma	
		detected m6A peaks		m6A methylated RRACH	detected RRACH	Averaged reads length, bp	m6A frequency	Grade	Survival
ENSG00000177822	TENM3-AS1	2		5	6	1137	227	no data	
ENSG00000197536	IRF1-AS1	1		6	8	807	135	no data	
ENSG00000204682	MIR1915HG	1		20	28	981	49	no data	
ENSG00000215386	MIR99AHG	1		2	4	890	445	no data	
ENSG00000222041	CYTOR (LINC00152)	2*		3	5	688	229	↑	↓
ENSG00000223768	LINC00205	3		7	11	1626	232	↓	
ENSG00000225733	FGD5-AS1	1		7	19	1567	224	↓	–
ENSG00000228794	LINC01128	1		19	34	2048	108	no data	
ENSG00000231721	LINC-PINT	2		9	25	1566	174	no data	
ENSG00000233016	SNHG7	1*		5	9	957	192	↓	–
ENSG00000233384	CNIH3-AS2	1		3	7	782	261	no data	
ENSG00000233621	LINC01137	1		10	14	1174	117	no data	
ENSG00000234456	MAGI2-AS3	2		5	15	1176	235	–	–
ENSG00000237232	ZNF295-AS1	1*		6	7	824	137	↑	↓
ENSG00000242125	SNHG3	1		3	11	998	333	↑	
ENSG00000245532	NEAT1	1		17	43	1306	77	↑	↓
ENSG00000246560	UBE2D3-AS1	1		6	11	521	88	no data	
ENSG00000251022	THAP9-AS1	1		30	63	1865	62	–	↓
ENSG00000251562	MALAT1	3		14	36	924	66	–	↓
ENSG00000253161	LINC01605	1		8	15	1381	173	no data	
ENSG00000260032	NORAD	1		19	46	1422	75	no data	
ENSG00000261455	LINC01003	1		9	16	1069	119	–	–
ENSG00000261801	LOXL1-AS1	2		3	3	946	315	↑	↓
ENSG00000267100	ILF3-AS1 (ILF3-DT)	1		4	11	1187	297	↓	↑

Note: ‘*’ – peak identified in MeRIP-seq did not map to gene body of annotated lncRNA. RRACH – motif where m6A resides (R can be either A or G, and H can be A, C or U). m6A overlap with RRACH motif provides additional proof for precise m6A. The arrow ‘↑’ indicates significantly increased lncRNA expression in higher-grade gliomas, while ‘↓’ – significantly decreased lncRNA levels in high-grade gliomas. The arrow ‘↑’ under the ‘Survival’ column indicates direct significant association with patients’ survival (higher expression – better survival prognosis), while ‘↓’ – opposite significant associations. The long dash ‘ – ’ indicates no significant association between lncRNA levels and glioma grade or patient survival. Indication ‘no data’ means there are no records of the gene in the Gliovis database.

Next, we decided to analyse m6A modification and m6A peaks identification features in those 24 overlapping lncRNA molecules. dRNA-seq in total identified 220 m6A modified sites out of 447 total sites (~49% modification rate) (Table 4). While MeRIP-seq only identified 33 m6A peaks (Table 4). It translates to 6–7 m6A modifications per m6A peak detected with MeRIP-seq.

We also investigated m6A modification frequency in identified lncRNAs using precise location of m6As from dRNA-seq. We decided to use an average read length rather than actual length of the molecule, because sequencing reads do not always cover the whole length of the molecule. We investigated 24 lncRNA molecules and found a tendency for different m6A modification frequency.

Modification frequency is interpreted as an average m6A occurrence interval in each molecule. The m6A modification occurrence varied from 49 to 445 nt in the list of modified 24 lncRNAs (overlapping between MeRIP-seq and dRNA-seq). The highest modification frequency was detected in *MIR1915HG, THAP9-AS1, MALAT1, NORAD1, NEAT1*, and *UBE2D3-AS1* – m6A every: 49, 62, 66, 75, 77, and 88 nt, respectively. While *MIR99AHG, SNHG3, LOXL1-AS1, ILF3-DT*, and *CNIH3-AS2* showed the lowest m6A modification frequency, accordingly m6A every 445, 333, 315, 297, and 261 nt. The rest of the lncRNAs showed intermediate m6A modification frequency (108–235 nt); see Table 4.

### dRNA-seq read coverage analysis of long and short lncRNAs

**Figure 5.**
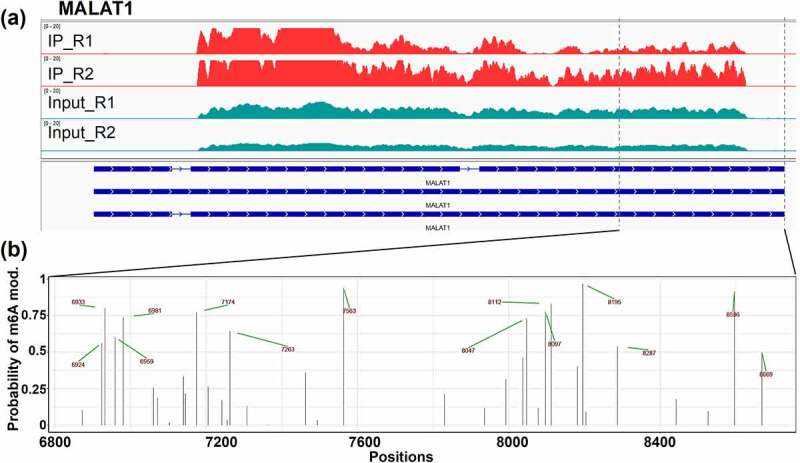
Illustration of identified *MALAT1* m6A modifications overlap in (a) MeRIP-seq and (b) Nanopore dRNA-seq. IP_R1/2 indicate MeRIP-seq [m6A] replicates; Input_R1/2 indicate MeRIP-seq [expression] replicates. Blue horizontal lines illustrate *MALAT1* transcripts. Black vertical lines represent adenines within RRACH motifs. Green arrows and red numbers indicate the exact position of m6A modifications. Panel (a) generated with IGV (Integrative Genomics Viewer) gene browser.

While MeRIP-seq data provided information about molecules methylation status, it did not produce desired resolution in terms of m6A peak calling; often peak start and end would overlap and preclude identification of the precise peak count. An example of *MALAT1* lncRNA MeRIP-seq m6A peaks are presented (Figure 5a). Meanwhile, dRNA-seq captured m6A modifications are mapped with more detail and precision (Figure 5b). While MeRIP-seq identified high m6A enrichment in 5’ region, with lower-intensity m6A signal towards the end of the transcript (Figure 5a), dRNA-seq showed high levels of m6A modifications (14 modified RRACH motifs) in the 3’ region (Figure 5b). Lack of full-length coverage for *MALAT1* lncRNA by dRNA-seq precluded identification of m6A modification status of 5’ region. Despite that dRNA-seq only resulted in data on the 3’ region of *MALAT1*, and its resolution greatly outperformed the information that MeRIP-seq provided for the full-length gene.

**Figure 6.**
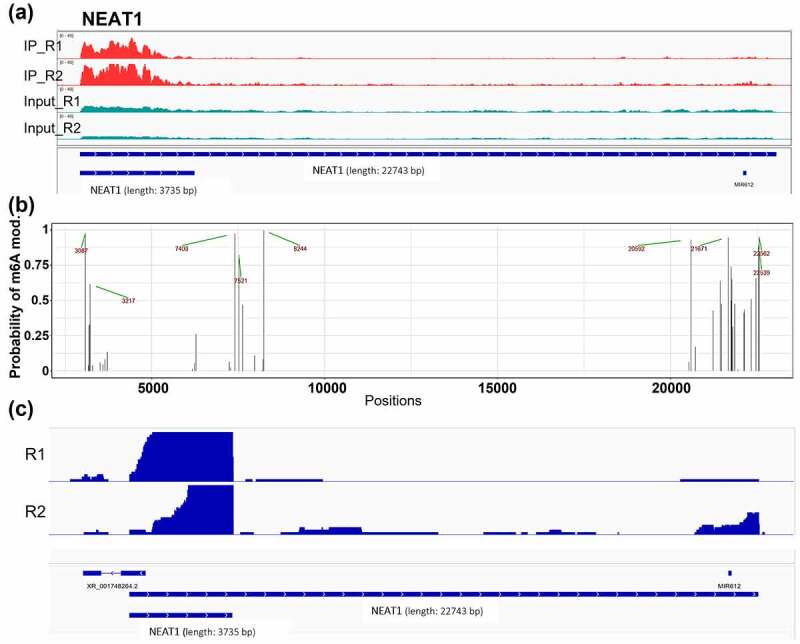
*NEAT1* m6A modifications and read distribution detected by (a) MeRIP-seq, (b) dRNA-seq. (c) *NEAT1* dRNA-seq read distribution throughout *NEAT1* gene body. In panel (a) red colour indicates MeRIP-seq [m6A] peaks from both replicates and teal colour – MeRIP-seq [expression] peaks from both replicates. Black vertical lines in panel (c) represent adenines within RRACH motifs. Green arrows and red numbers indicate the exact position of m6A modifications. In panel (c) R1/2 indicate dRNA-seq replicates and blue horizontal lines illustrate *NEAT1* transcripts. Panel (a) and (b) generated with IGV (Integrative Genomics Viewer) gene browser.

In contrast to the long lncRNA, *MALAT1*, analysis of short lncRNAs (*CYTOR, IRF1-AS1* and *ZNF295-AS1*), with lengths close to the median read length of dRNA-seq (~900 nt), showed dRNA-seq coverage of the entire molecule (**Figure S1 and S2**). lncRNAs lengths are listed in (**Table S2**). Further analysis of long lncRNA molecules like *MALAT1* and *NEAT1* revealed identical pattern of m6A identification in 3’ but not in 5’ prime region due to dRNA-seq difficulties to obtain enough coverage for 5’ region of long (> 1000 nt) RNA molecules. This difficulty is due to the fragility of RNA molecules, during RNA isolation and sequencing library preparation. Interestingly, two high-coverage dRNA-seq locations were observed in *NEAT1* gene. First location is, as expected, located in 3’ region; however, the second location is present in 5’ region. The latter location can only be explained by looking in additional transcripts of *NEAT1* gene, which indeed starts in the same region as the second *NEAT1* read peak (Figure 6).

### Gliovis expression analysis of m6A modified lncRNAs

After setting the list of lncRNA, in which m6A modifications were identified by both MeRIP-seq and dRNA-seq methods, it was decided to search open-access databases to evaluate if identified molecules were already found to be associated with pathology. Since high throughput epi-transcriptome research is still limited by available methods we were not able to find any information in databases on lncRNAs m6A modifications in glioma specimens. Thus, we decided to check whether the expression changes of highly m6A modified lncRNAs differ between glioma samples; for this purpose we used the analysis at Gliovis. The association between the expression of identified lncRNAs and glioma malignancy, as well as patients’ survival, was examined using TCGA and CCGA datasets stored in the *Gliovis* database [32]. *CYTOR, NEAT1, LOXL1-AS1*, and *ZNF295-AS1* were significantly associated with glioma grade and survival. The increased expression of these lncRNAs is found in high-grade gliomas, and is associated with poor survival prognosis. High expression of *ILF3-AS1* was associated with low tumour grade and longer survival (Table 4). Significantly decreased levels of *LINC00205, FGD5-AS1,* and *SNHG7* were characteristic for high-grade gliomas as compared to lower-grade tumours, while *SNHG3* showed opposite associations. Gene expression information of 11 screened lncRNAs were not available in the Gliovis database (Table 4).

## Discussion

Our results support potential edge for dRNA-seq over MeRIP-seq when analysing specific m6A modifications in lncRNAs. This is of particular importance as lncRNAs and ncRNAs contain more methylation sites than mRNAs. We provide information about 220 precise m6A modifications or 33 m6A peaks in 24 lncRNA molecules of U87-MG cell line, and m6A methylation frequency in those lncRNAs, listing most and least m6A modified lncRNAs.

dRNA-seq uses first-strand, unamplified cDNA for sequencing, whereas MeRIP-seq uses PCR-amplified cDNA. This results in a high coverage and detection rate of lncRNAs using MeRIP-seq approach. Although the difference in mapped read quantity between these methods may not the best parameter to compare their overall performance. MeRIP-seq reads are ~20 times shorter than dRNA-seq; therefore, the chance of detecting multiple molecules is higher than sequencing a long, continuous molecule, as done in dRNA-seq. However, these long, continuous molecules are in their native form and better represents their biological state by preserving accurate location of RNA modification and exon-exon junctions. In addition, dRNA-seq provides precise m6A location at a single nucleotide resolution. This enables an accurate analysis of the adenosine methylation status in a RRACH-rich transcriptomic regions, whereas the resolution given by the MeRIP-seq is not able to distinguish a specific adenosine methylation status if there are multiple RRACH motifs close to each other. Furthermore, RNA fragmentation step required in MeRIP-seq could potentially result in the underrepresentation of m6As close to the 3’ end of RNA. Since NGS-seq provides short reads and TTS shows similarity among transcripts [33,34], the RNA fragments from 3’ end mostly composed of polyA and fished-out by MeRIP assay are mostly unannotated and stay uncovered after data processing. In contrast, dRNA-seq starts to sequence RNA molecules from their 3’ end and is able to reveal m6A methylation status at 3’ end of transcripts, due to its ability to sequence long, continuous RNA fragments.

Low expression levels of lncRNAs coupled with RNA brakes during library preparation, and moderate erroneous of dRNA-seq itself could lead to an underrepresentation of 5’ regions of lncRNAs, as illustrated by the *MALAT1* example. However, it is not a critical factor for highly expressed or short RNAs up to ~1000 bp. One of the possibilities to overcome low coverage is an enrichment for specific lncRNA molecules during library preparation, modifying the reverse transcriptase adapter (RTA) by incorporating a lncRNA-specific anti-sense sequence. Sequencing of enriched lncRNA molecules should allow to obtain information of m6A status in full length of the transcript, and in high coverage.

The need of single nucleotide resolution is further improved by the latest’s studies. Z.-X. Li et al., functionally verified the importance of specific lncRNA m6A modification for nasopharyngeal carcinoma growth and metastasis [35]. This finding could have never been achieved with MeRIP-seq and regular Illumina RNA-seq. Therefore, despite higher base-calling error rate and previously mentioned limitations, dRNA-seq still edges MeRIP-seq for its suitability to study epi-transcriptomic changes in a specific part of the non-coding transcriptome. The advantage of dRNA-seq is particularly noticeable, when RNA modifications are explored in a set of specific transcripts. The long reads outputted by dRNA-seq enable the distinguishment of two very similar transcripts, when there is a high percentage of exon overlap between them. Current study revealed that despite MeRIP-seq identified ~14 times more lncRNA transcripts than dRNA-seq, it only revealed 2.8 times more m6A modified lncRNAs as compared to dRNA-seq. Such a data indicates dRNA-seq advantage for higher resolution of identifying methylated adenines in RNAs. Such a feature could be explained by dRNA-seq ability to identify single m6A at single RNA transcript, while MeRIP m6A peak from a single pulled-out fragment would be insignificant if at all. Antibody-based approaches may underestimate the number of m6A sites [36]. This is because MeRIP consist of many stages and at some stages m6A mark could be lost due to ruthless thermal conditions; moreover, at each stage portion of RNA material is lost [37,38].

Moreover, dRNA-seq provides possibility to detect other chemical modifications in transcriptome like pseudo-uridine, m5C, inosine, etc. These modifications could be mapped at a single nucleotide resolution in all expressed transcripts and provide an overall profile of the epi-transcriptome. Even though the lack of bioinformatic tools for other epi-transcriptomic RNA modifications is currently a limiting step, dRNA-seq data could be reanalysed with novel tools upon development. Specifically in our context of m6A identification in lncRNAs the lack of annotated ncRNAs is an issue, especially if MeRIP-seq is used, and secondly, unlike coding genes, lncRNA genes often overlap [39]. Overlapping could lead to discovery of false positives; this can be easily overcome by manual screening of the data in the MeRIP-seq case. Especially if the list of modified lncRNAs is small.

We identified 24 overlapping lncRNA between MeRIP-seq and dRNA-seq. We suggest that identified molecules were modified in similar regions, even though dRNA-seq provide a more precise location. Furthermore, we hypothesize that this list predominantly consists of molecules with modifications in 3’ region for previously explained, 5’ region underrepresentation, reasons. We successfully mapped 14 m6A modifications at a single nucleotide resolution within *MALAT1* 3’ region which provided more information about *MALAT1* m6A status than MeRIP-seq from the whole gene. Additionally, we were able to calculate the frequency of m6A modifications in lncRNAs, which provides a better understanding of the m6A status, than total number of m6A modifications per molecule. Based on mRNA research done by B. Liu et al., where authors revealed that m6A modification is affecting secondary structures of mRNAs [40,41], we theorize that m6A frequency could affect the secondary structure of lncRNAs, leading to its altered function. We believe that higher m6A frequency in lncRNAs can lead to its less flexible folding and weaken function; however, this must be proven by functional experiments.

Gliovis dataset analysis revealed that the expression levels of more than half lncRNAs that were detected as m6A modified using both methods were significantly associated with glioma malignancy grade or patient survival prognosis or both parameters. Taken all together it suggests that glioma pathogenesis and progression could be better explained by looking at combinatory analysis of transcriptomics and epi-transcriptomics rather than just transcriptomics alone. Future perspectives for m6A modification frequency could be further explored, and it may provide a better indication of cancer malignancy than global m6A level or expression levels of m6A machinery. Specific lncRNA targets and precise locations of m6A modifications detected in current research could be useful for future studies to validate the suitability of identified molecules for glioma screening and potential novel therapeutics, especially as CRISPR RNA editing already enables location-specific m6A installation and removal. This approach enables functional studies for m6A effects, and later could be repaired by genome editing using CRISPR base editing or CRISPR prime editing for removing undesired RRACH motifs.

Current studies have shown that the abnormal expression of m6A-related genes is closely associated with the tumorigenesis and progression of glioma [42,43]. However, most of these studies only focus on RNA expression of m6A ‘writers’ (methyltransferases), ‘readers’ (signal transducers), and ‘erasers’ (demethylases), but not to their specific targets. LncRNA molecules in most cases contain more than one adenine that can be modified. Very little is known about the functions of specific lncRNAs, their secondary structure and possible interactions with DNA, mRNA, miRNA, or proteins. Even less is known about how m6A modifications occurring at a specific location on a lncRNA molecule can influence the functions of that lncRNA.

In this study, we attempted to determine which lncRNA molecules potentially undergo changes of m6A modifications in gliomas. The list of 24 lncRNA molecules that undergo m6A modifications in the glioblastoma cell line allows us to assume that these modifications are possibly important for the growth and development of gliomas. We believe that higher m6A frequency in lncRNAs can lead to its less flexible folding and weaken function; however, this must be proven by functional experiments. In summary, we provide a high accuracy list of m6A modification locations in 24 lncRNA molecules of U87-MG cells.

Extensive studies in human glioblastoma samples are necessary to confirm the importance of identified lncRNAs m6A modifications in the processes of gliomagenesis, response to treatment, or prognosis. Since the amounts of tissue material are very limited when working with human or animal specimens, and the large amounts of RNA (accordingly, tissue) are required for the m6A detection assays, especially MeRIP-seq, it would be very difficult, if at all possible, to perform transcriptome-wide m6A methylation analysis applying both MeRIP-seq and dRNA-seq assays on the tissue from the same individual. After examining the advantages and disadvantages of m6A detection methods applying U87 cell lines, we are able to conclude which method would be more appropriate for m6A analysis in tissue studies.

Our data suggests that dRNA-seq is more suitable for studying chemical modifications of specific lncRNAs, rather than MeRIP-seq when single nucleotide resolution and m6A frequency are required or when the region of interest is in 3’ region. On the other hand, MeRIP-seq is advantageous when screening of novel or lowly expressed molecules carrying m6A modification is intended to be done. We conclude that MeRIP-seq is more suitable for an initial m6A studies, due to its higher lncRNA coverage, whereas dRNA-seq is most useful when a specific list of m6A modified lncRNA molecules are already known, and a more in-depth analysis of m6A quantity and precise location is of interest.

## Supplementary Material

Supplemental Material

## Data Availability

The datasets generated and analysed during the current study are available in the Sequence Read Archive SRA repository, https://dataview.ncbi.nlm.nih.gov/object/PRJNA917040?reviewer=r52jnkdi9kh2e653p9p7bi20qe

## References

[1] COHN WE, VOLKIN E. Nucleoside-5'-phosphates from ribonucleic acid. Nature. 1951 Mar;167(4247):483–14.

[2] Boccaletto P, Machnicka MA, Purta E, et al. MODOMICS: a database of RNA modification pathways. 2017 update. Nucleic Acids Res. 2018 Jan;46(D1):D303–D307. 29106616 10.1093/nar/gkx1030 PMC5753262

[3] Jia G, Fu Y, Zhao X, et al. N6-Methyladenosine in nuclear RNA is a major substrate of the obesity-associated FTO. Nat Chem Biol. 2011 Dec;7(12):885–887. 22002720 10.1038/nchembio.687 PMC3218240

[4] Zheng G, Dahl J, Niu Y, et al. ALKBH5 is a mammalian RNA demethylase that impacts RNA metabolism and mouse fertility. Mol Cell. 2013 Jan;49(1):18–29. 23177736 10.1016/j.molcel.2012.10.015 PMC3646334

[5] Dominissini D, Moshitch-Moshkovitz S, Salmon-Divon M, et al. Transcriptome-wide mapping of N6-methyladenosine by m6A-seq based on immunocapturing and massively parallel sequencing. Nat Protoc. 2013 Jan;8(1):176–189. 23288318 10.1038/nprot.2012.148

[6] Meyer KD, Saletore Y, Zumbo P, et al. Comprehensive analysis of mRNA methylation reveals enrichment in 3' UTRs and near stop codons. Cell. 2012 Jun;149(7):1635–1646. 22608085 10.1016/j.cell.2012.05.003 PMC3383396

[7] Schwartz S, Mumbach M, Jovanovic M, et al. Perturbation of m6A writers reveals two distinct classes of mRNA methylation at internal and 5' sites. Cell Rep. 2014 Jul;8(1):284–296. 24981863 10.1016/j.celrep.2014.05.048 PMC4142486

[8] Xu C, Wang X, Liu K, et al. Structural basis for selective binding of m6A RNA by the YTHDC1 YTH domain. Nat Chem Biol. 2014 Nov;10(11):927–929. 25242552 10.1038/nchembio.1654

[9] Fu Y, Jia G, Pang X, et al. FTO-mediated formation of N6-hydroxymethyladenosine and N6-formyladenosine in mammalian RNA. Nat Commun. 2013 Jun;4(1). DOI:10.1038/ncomms2822. PMC3658177 23653210

[10] Roundtree IA, Evans ME, Pan T, et al. Dynamic RNA modifications in gene expression regulation. Cell. 2017 Jun;169(7):1187–1200. 28622506 10.1016/j.cell.2017.05.045 PMC5657247

[11] Frye M, Harada BT, Behm M, et al. RNA modifications modulate gene expression during development. Science. 1979 , Sep. 2018;361(6409). DOI:10.1126/science.aau1646. PMC6436390 30262497

[12] Hsu PJ, Shi H, He C. Epitranscriptomic influences on development and disease. Genome Biol. 2017 Dec;18(1):DOI:10.1186/s13059-017-1336-6 PMC5654102 29061143

[13] Liu H, Begik, O, Lucas, MC, et al. Accurate detection of m6A RNA modifications in native RNA sequences. Nat Commun. 2019 Dec;10(1). DOI:10.1038/s41467-019-11713-9. PMC6734003 31501426

[14] Coker H, Wei G, Moindrot B, et al. The role of the Xist 5’ m6A region and RBM15 in X chromosome inactivation. Wellcome Open Res. 2020 Feb;5:31. 32258426 10.12688/wellcomeopenres.15711.1 PMC7097882

[15] Ma S, Chen C, Ji X, et al. The interplay between m6A RNA methylation and noncoding RNA in cancer. J Hematol Oncol. 2019 Dec;12(1). DOI:10.1186/s13045-019-0805-7. PMC6874823 31757221

[16] Ariel F, Lucero L, Christ A, et al. R-loop mediated trans action of the APOLO long noncoding RNA. Mol Cell. 2020 Mar;77(5):1055–1065.e4. 31952990 10.1016/j.molcel.2019.12.015

[17] Gupta RA, Shah N, Wang KC, et al. Long non-coding RNA HOTAIR reprograms chromatin state to promote cancer metastasis. Nature. 2010 Apr;464(7291):1071–1076. 20393566 10.1038/nature08975 PMC3049919

[18] Zhang K, Han X, Zhang Z, et al. The liver-enriched lnc-LFAR1 promotes liver fibrosis by activating TGFβ and Notch pathways. Nat Commun. 2017 Dec;8(1). DOI:10.1038/s41467-017-00204-4. PMC5529527 28747678

[19] Zhang Y, Pitchiaya S, Cieślik M, et al. Analysis of the androgen receptor–regulated lncRNA landscape identifies a role for ARLNC1 in prostate cancer progression. Nat Genet. 2018 Jun;50(6):814–824. 29808028 10.1038/s41588-018-0120-1 PMC5980762

[20] Linder B, V Grozhik A, Olarerin-George AO, et al. Single-nucleotide-resolution mapping of m6A and m6Am throughout the transcriptome. Nat Methods. 2015 Aug;12(8):767–772. 26121403 10.1038/nmeth.3453 PMC4487409

[21] Patil DP, Chen C-K, Pickering BF, et al. m6A RNA methylation promotes XIST-mediated transcriptional repression. Nature. 2016 Sep;537(7620):369–373. 27602518 10.1038/nature19342 PMC5509218

[22] Zhou KI, Parisien M, Dai Q, et al. N6-methyladenosine modification in a long noncoding RNA hairpin predisposes its conformation to protein binding. J Mol Biol. 2016 Feb;428(5):822–833. 26343757 10.1016/j.jmb.2015.08.021 PMC4779075

[23] Uphoff CC, Drexler HG. Detection of mycoplasma contamination in cell cultures. Curr Protoc Mol Biol. 2014 Apr;106(1):DOI:10.1002/0471142727.mb2804s106 24733240

[24] Broad Institute, “Picard Toolkit.” Broad Institute, Boston, 2019. cited: Oct. 17, 2022. [Online 2022 Oct 17: https://broadinstitute.github.io/picard/

[25] Kim D, Paggi JM, Park C, et al. Graph-based genome alignment and genotyping with HISAT2 and HISAT-genotype. Nat Biotechnol. 2019 Aug;37(8):907–915. 31375807 10.1038/s41587-019-0201-4 PMC7605509

[26] Ewels PA, Peltzer A, Fillinger S, et al. The nf-core framework for community-curated bioinformatics pipelines. Nat Biotechnol. 2020 Mar;38(3):276–278. 32055031 10.1038/s41587-020-0439-x

[27] Li H. Minimap2: pairwise alignment for nucleotide sequences. Bioinformatics. 2018 Sep;34(18):3094–3100. 29750242 10.1093/bioinformatics/bty191 PMC6137996

[28] Danecek P, Bonfield JK, Liddle J, et al. Twelve years of SAMtools and BCFtools. Gigascience. 2021 Jan;10(2). DOI:10.1093/gigascience/giab008. PMC7931819 33590861

[29] Wang L, Wang S, Li W. RSeQC: quality control of RNA-seq experiments. Bioinformatics. 2012 Aug;28(16):2184–2185. 22743226 10.1093/bioinformatics/bts356

[30] Wang L, Nie J, Sicotte H, et al. Measure transcript integrity using RNA-seq data. BMC Bioinformatics. 2016 Dec;17(1):58. 26842848 10.1186/s12859-016-0922-z PMC4739097

[31] de Coster W, D’Hert S, Schultz DT, et al. NanoPack: visualizing and processing long-read sequencing data. Bioinformatics. 2018 Aug;34(15):2666–2669. 29547981 10.1093/bioinformatics/bty149 PMC6061794

[32] Bowman RL, Wang Q, Carro A, et al. GlioVis data portal for visualization and analysis of brain tumor expression datasets. Neuro Oncol. 2017;19(1):139–141. 28031383 10.1093/neuonc/now247 PMC5193031

[33] Kuo RI, Tseng E, Eory L, et al. Normalized long read RNA sequencing in chicken reveals transcriptome complexity similar to human. BMC Genomics. 2017 Apr;18(1):DOI:10.1186/S12864-017-3691-9 PMC5404281 28438136

[34] Trapnell C, Williams BA, Pertea G, et al. Transcript assembly and quantification by RNA-Seq reveals unannotated transcripts and isoform switching during cell differentiation. Nat Biotechnol. 2010 May;28(5):511–515. 20436464 10.1038/nbt.1621 PMC3146043

[35] Li Z-X, Zheng Z-Q, Yang P-Y, et al. WTAP-mediated m6A modification of lncRNA DIAPH1-AS1 enhances its stability to facilitate nasopharyngeal carcinoma growth and metastasis. Cell Death Differ. 2022 Jan. DOI:10.1038/s41418-021-00905-w. PMC9177844 34999731

[36] Garcia-Campos MA, Edelheit S, Toth U, et al. Deciphering the ‘m6A code’ via antibody-independent quantitative profiling. Cell. 2019 Jul;178(3):731–747.e16. 31257032 10.1016/j.cell.2019.06.013

[37] Yang H, Lam SL. Effect of 1-methyladenine on thermodynamic stabilities of double-helical DNA structures. FEBS Lett. 2009 May;583(9):1548–1553. 19376116 10.1016/j.febslet.2009.04.017

[38] McIntyre ABR, Gokhale NS, Cerchietti L, et al. Limits in the detection of m6A changes using MeRIP/m6A-seq. Sci Rep. 2020 Dec;10(1):DOI:10.1038/S41598-020-63355-3 PMC7170965 32313079

[39] Ning Q, Li Y, Wang Z, et al. The evolution and expression pattern of human overlapping lncRNA and protein-coding gene pairs. Sci Rep. 2017 Mar;7(1):DOI:10.1038/srep42775 PMC5366806 28344339

[40] Liu B, Feo T, Harvey TA, et al. A potentially abundant junctional RNA motif stabilized by m6A and Mg2+. Nat Commun. 2018;9(1):1–10. 30018356 10.1038/s41467-018-05243-z PMC6050335

[41] Liu N, Zhou KI, Parisien M, et al. N6-methyladenosine alters RNA structure to regulate binding of a low-complexity protein. Nucleic Acids Res. 2017 Jun;45(10):6051–6063. 28334903 10.1093/nar/gkx141 PMC5449601

[42] Tu Z, Wu L, Wang P, et al. N6-methylandenosine-related lncRNAs are potential biomarkers for predicting the overall survival of lower-grade glioma patients. Front Cell Dev Biol. 2020 Jul;8. DOI:10.3389/FCELL.2020.00642 PMC7390977 32793593

[43] Cong P, Wu T, Huang X, et al. Identification of the role and clinical prognostic value of target genes of m6A RNA methylation regulators in glioma. Front Cell Dev Biol. 2021 Sep;9. DOI:10.3389/FCELL.2021.709022 PMC8473691 34589481

