# Supplementary figures and images for: Identification and comparison of m6A modifications in glioblastoma non-coding RNAs with MeRIP-seq and Nanopore dRNA-seq
Source: Epigenetics. 2023 Jan 3;18(1):2163365. doi: 10.1080/15592294.2022.2163365 (PMC9980576; doi:10.1080/15592294.2022.2163365)

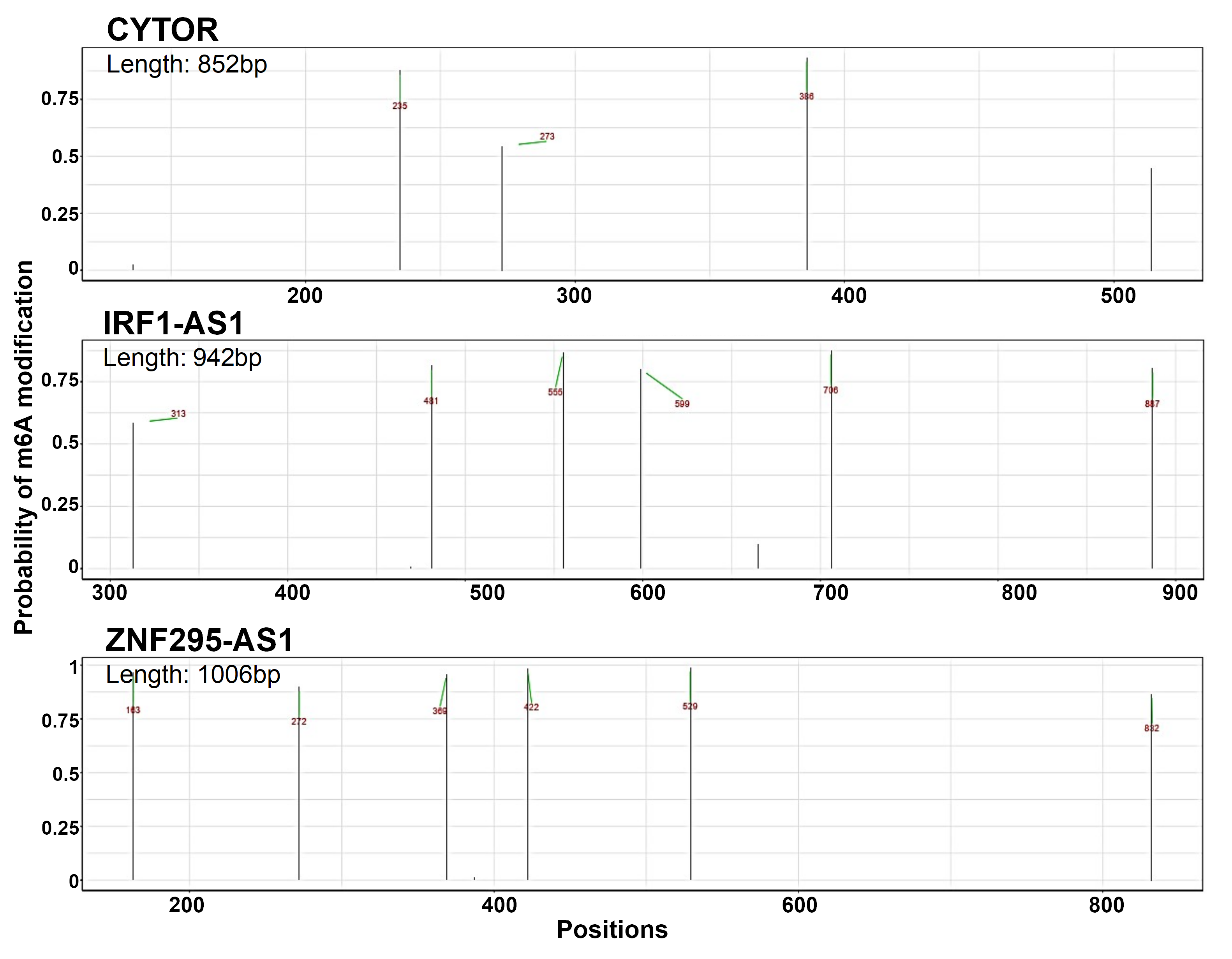

Supplement: Supplemental Material [file KEPI_A_2163365_SM4029.zip › Supplementary files/SF1.tif]

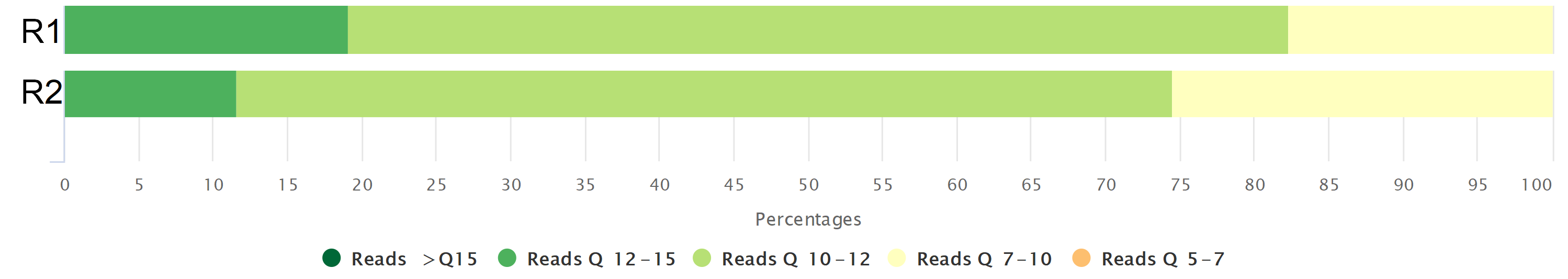

Supplement: Supplemental Material [file KEPI_A_2163365_SM4029.zip › Supplementary files/SF2.tif]
